# Association between remnant cholesterol–based lipid indicators and testosterone deficiency in middle-aged and older men: a cross-sectional study

**DOI:** 10.3389/fendo.2026.1782710

**Published:** 2026-04-01

**Authors:** Xingliang Feng, Guoyang Zhang, Yu Liu, Zhenlei Zha, Wei Zhang, Qianfeng Zhuang, Hu Zhao, Yangyang Mei

**Affiliations:** 1 Department of Urology, The Third Affiliated Hospital of Soochow University, Changzhou, Jiangsu, China; 2 Department of Urology, Jiangyin People’s Hospital, the Jiangyin Clinical College of Xuzhou Medical University, Jiangyin, Jiangsu, China; 3 Department of Urology, The Second Affiliated Hospital of Anhui Medical University, Hefei, China

**Keywords:** cross-sectional study, lipid metabolism, remnant cholesterol, systemic inflammation, testosterone deficiency

## Abstract

**Background:**

Remnant cholesterol (RC), representing cholesterol carried in triglyceride-rich lipoproteins, has emerged as an important marker of residual cardiometabolic risk. Recent population-based studies have suggested an association between RC and testosterone deficiency (TD); however, evidence from real-world clinical populations and the potential involvement of inflammatory pathways remains limited.

**Methods:**

This multicenter cross-sectional study enrolled 387 men aged 50–75 years from urology and andrology outpatient clinics at three hospitals between December 2024 and December 2025. Testosterone deficiency was diagnosed according to American Urological Association guidelines, defined as total testosterone <300 ng/dL accompanied by relevant clinical symptoms. Fasting blood samples were obtained to assess lipid profiles, sex hormones, and inflammatory markers. Remnant cholesterol was calculated as total cholesterol minus HDL-C and LDL-C. Multivariable logistic regression analyses were used to examine the association between RC and TD. Receiver operating characteristic (ROC) curve analysis evaluated the discriminative ability of RC compared with traditional lipid parameters. Mediation analyses were performed using the Hayes PROCESS macro (model 4) with 5,000 bootstrap resamples to assess the potential mediating roles of high-sensitivity C-reactive protein (hs-CRP) and interleukin-6 (IL-6).

**Results:**

Among the 387 participants, 183 were diagnosed with TD. Higher RC levels were significantly associated with increased odds of TD after adjustment for demographic factors, lifestyle variables, metabolic comorbidities, medication use, and triglyceride levels (adjusted OR per 1 mmol/L increase: 3.33, 95% CI: 2.35–4.71). When categorized into quartiles, RC showed a clear dose–response association with TD (all P < 0.001). In ROC analysis, RC demonstrated moderate discriminative ability for identifying TD (AUC = 0.733, 95% CI: 0.684–0.782), outperforming total cholesterol (AUC = 0.696), triglycerides (AUC = 0.634), and LDL-C (AUC = 0.605). Mediation analyses indicated that hs-CRP and IL-6 partially mediated the association between RC and TD, accounting for 23.57% and 16.49% of the total effect, respectively.

**Conclusions:**

In this multicenter cross-sectional study, elevated remnant cholesterol was associated with a higher prevalence of TD and showed superior discriminative performance compared with traditional lipid parameters. Longitudinal studies are needed to clarify temporal relationships and underlying mechanisms.

## Introduction

Testosterone deficiency (TD) is a prevalent endocrine disorder among middle-aged and older men and is clinically characterized by both biochemical evidence of low testosterone and the presence of relevant symptoms and/or signs (1). Testosterone, a principal androgen in male reproductive physiology, is integral to the development of sexual desire, erectile function, and secondary sexual characteristics (2, 3). According to the American Urological Association (AUA) guideline, the diagnosis of TD requires consistently low total testosterone levels together with selected clinical manifestations (4). With population aging, TD has become increasingly common in urology and andrology practice and is associated with impaired sexual function, reduced vitality, and adverse cardiometabolic profiles (5–7).

Increasing evidence indicates a close interplay between androgen status and lipid metabolism (8). Men with lower testosterone levels often present with unfavorable lipid profiles and other metabolic disturbances, whereas dyslipidemia and metabolic syndrome may, in turn, be linked to impaired gonadal function (3, 9). Traditional lipid parameters—including total cholesterol (TC), triglycerides (TG), low-density lipoprotein cholesterol (LDL-C), and high-density lipoprotein cholesterol (HDL-C)—have been examined in relation to testosterone levels in prior studies (10–12); however, findings have been inconsistent, and conventional lipid indices may not sufficiently capture the atherogenic lipid fractions most relevant to metabolic–endocrine dysfunction. In recent years, remnant cholesterol (RC), defined as the cholesterol content of triglyceride-rich lipoprotein remnants and calculated as TC minus HDL-C and LDL-C, has emerged as a novel and clinically accessible lipid indicator (13). RC reflects the atherogenic burden of remnant lipoproteins (14, 15) and has been shown to be closely associated with cardiometabolic disorders, including insulin resistance, metabolic syndrome, and cardiovascular disease (CVD) (16, 17). Importantly, these conditions overlap substantially with the pathophysiological milieu of TD, suggesting that RC may represent a biologically relevant link between dyslipidemia and impaired gonadal function.

Recently, large population-based studies using the National Health and Nutrition Examination Survey (NHANES) database have provided initial evidence supporting a relationship between RC and TD. In particular, our research group previously demonstrated that higher RC levels were independently associated with lower total testosterone concentrations and an increased risk of TD in U.S. adult men (18). Similar findings were subsequently corroborated in another NHANES-based analysis, reinforcing the robustness of the observed association at the population level (19). While these studies provide important epidemiological evidence, their reliance on secondary survey data imposes inherent limitations, including the absence of detailed clinical symptom assessment, limited inflammatory profiling, and potential misclassification of testosterone deficiency based solely on biochemical thresholds.

Moreover, the biological mechanisms linking elevated RC to testosterone deficiency remain incompletely understood. Chronic low-grade inflammation has been proposed as a plausible pathway, as remnant lipoproteins can promote inflammatory activation through endothelial dysfunction, macrophage uptake, and cytokine release (20, 21). Inflammatory mediators such as high-sensitivity C-reactive protein (hs-CRP) and interleukin-6 (IL-6) have been implicated in both metabolic dysregulation and suppression of Leydig cell steroidogenesis (22–24). However, whether systemic inflammation mediates, at least in part, the association between RC and TD has not been directly examined in clinically characterized populations.

Against this background, the present multicenter cross-sectional study aimed to investigate the association between RC and TD in middle-aged and older men recruited from urology and andrology outpatient clinics. Building upon prior NHANES-based findings, we sought to validate this relationship in a real-world clinical setting, where TD was diagnosed according to established guideline criteria incorporating both biochemical measurements and clinical features. Furthermore, we evaluated the discriminatory performance of RC compared with traditional lipid parameters and explored the potential mediating roles of hs-CRP and IL-6 in the RC–TD relationship. We hypothesized that elevated RC would be independently associated with a higher risk of TD and that systemic inflammation would partially mediate this association.

## Methods

This multicenter, cross-sectional study was conducted at The Third Affiliated Hospital of Soochow University, Jiangyin People’s Hospital, and The Second Affiliated Hospital of Anhui Medical University. The study aimed to assess the association between RC and TD among men aged 50–75 years. Participants were recruited from the urology and andrology outpatient clinics at these three hospitals between December 2024 and December 2025. Eligible participants provided written informed consent and underwent clinical evaluations, including measurements of serum testosterone and lipid profiles. The study protocol was approved by the local institutional ethics committees, and written informed consent was obtained from all participants prior to enrollment.

**Figure 1:**
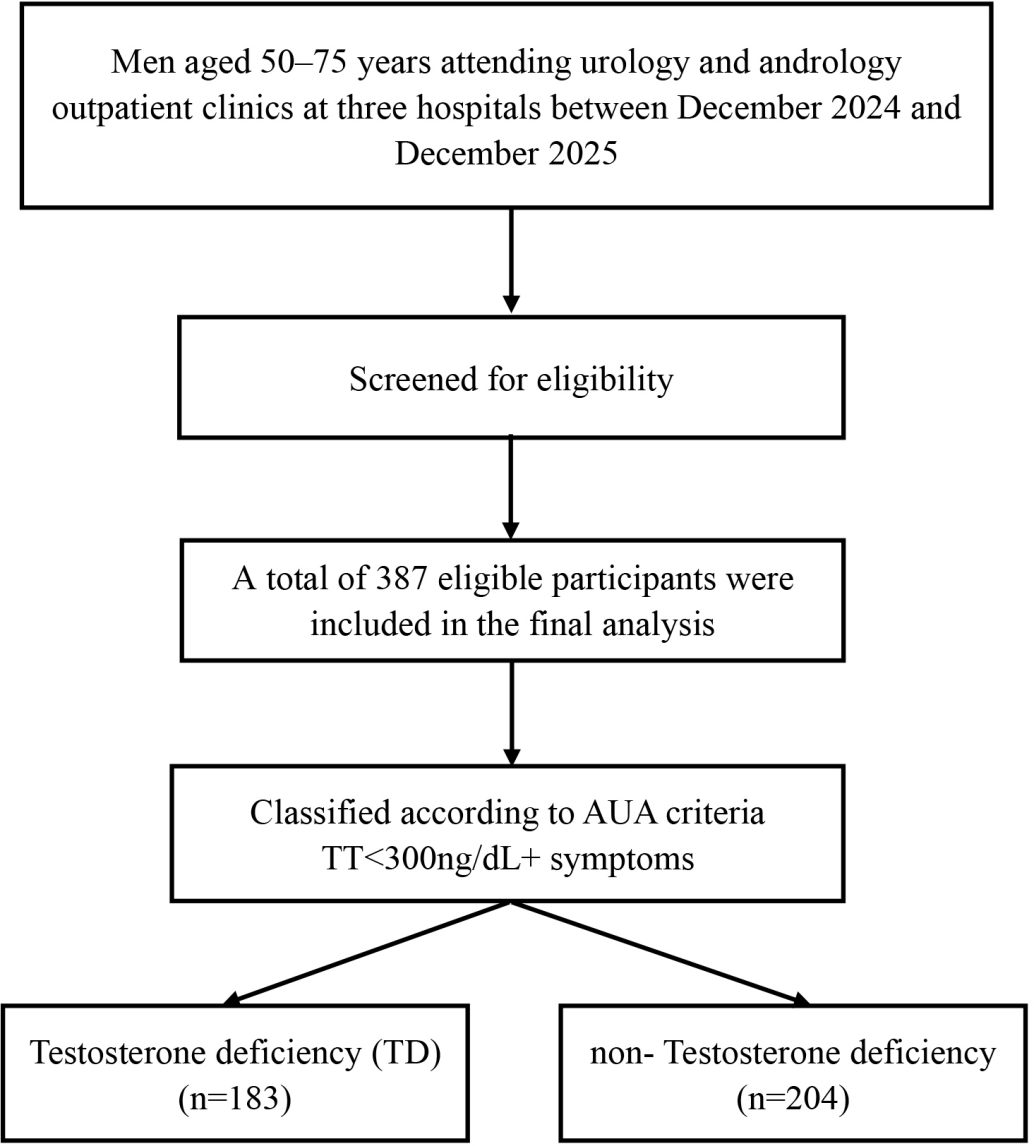
Flowchart illustrating the selection and classification of study participants.

The inclusion criteria were male patients aged 50–75 years who attended the participating clinics and provided informed consent. Patients were excluded if they were currently undergoing radiation therapy, using neuroblocking agents, antidepressants, or steroids, or receiving testosterone replacement therapy. Additionally, individuals with liver or thyroid diseases, gonadal dysfunction (such as primary hypogonadism), chronic renal failure, pituitary dysfunction, or chronic liver disease were also excluded from the study. The patient screening process is illustrated in **Figure 1**.

### Clinical and laboratory measurements

Demographic and clinical data were collected through patient self-reports and medical records. Age and body mass index (BMI) were measured as part of routine clinical evaluations. Medical comorbidities, including diabetes, and hypertension, were assessed through patient self-report and confirmed by medical records or prescription history. The diagnosis of hypertension was based on self-reported medical history of hypertension or the use of antihypertensive medications. Similarly, diabetes was diagnosed through patient self-report or confirmed by the use of antidiabetic medications. The use of lipid-lowering medications was also documented, including those for metabolic agents, antihyperlipidemic drugs, and HMG-CoA reductase inhibitors (statins).

After an overnight fasting period of at least 12 hours, venous blood samples were collected from all participants in the morning. The blood samples were processed and stored at −80 °C until further analysis. Laboratory analyses were performed following standardized procedures. TC and TG were measured using enzymatic methods, with commercial reagent kits. HDL-C and LDL-C were measured using direct enzymatic assays. Total testosterone (TT) was measured using chemiluminescent immunoassay (CLIA). hs-CRP and IL-6 levels were measured using CLIA. Sex hormone-binding globulin (SHBG), estradiol (E2), and free testosterone (FT) concentrations were also measured using CLIA methods. RC was calculated using the formula RC = TC − HDL-C − LDL-C.

TD was diagnosed according to the AUA guidelines (4), which define TD as TT levels below 300 ng/dL accompanied by relevant clinical symptoms, including reduced sexual desire, erectile dysfunction, fatigue, and decreased muscle mass.

### Statistical analysis

Continuous variables were tested for normality using the Shapiro-Wilk test and presented as mean ± standard deviation (SD) for normally distributed data or median (interquartile range, IQR) for non-normally distributed data. Categorical variables were expressed as counts and percentages. Comparisons between groups (TD vs. Without TD) were conducted using the independent-samples t-test or Mann–Whitney U test for continuous variables and the chi-square test for categorical variables.

The relationship between RC and TD was assessed using multivariable logistic regression analysis. The primary independent variable, RC, was treated as both a continuous variable and categorized into quartiles (Q). To explore the potential mediating role of inflammatory markers in the association between RC and TD, a multiple mediation analysis was performed using the SPSS PROCESS macro developed by Hayes (model 4). This model allowed for the estimation of the indirect (mediated) effects of hs-CRP and IL-6 in the relationship between RC and TD. The bootstrap resampling method (5000 iterations) was used to estimate the indirect effects.

Receiver operating characteristic (ROC) curves were generated to evaluate the discriminative ability of RC, TC, LDL-C, and TG for identifying TD. The area under the curve (AUC) was calculated to compare the predictive performance of RC with traditional lipid parameters. The optimal cut-off points for each lipid measure were determined using the Youden index. All statistical analyses were conducted using IBM SPSS Statistics version 26.0 (IBM Corp., Armonk, NY, USA), and a two-tailed P value of less than 0.05 was considered statistically significant.

## Results

### Baseline characteristics of the study population

Baseline characteristics are presented in **Table 1**. Among the 387 men included, 183 were diagnosed with TD. Compared with participants without TD, men with TD were older (63.03 ± 4.80 vs. 61.69 ± 5.23 years, P = 0.009) and had a higher BMI (28.50 ± 2.67 vs. 27.67 ± 2.77 kg/m², P = 0.003). Smoking, alcohol consumption, and regular exercise did not differ significantly between groups. The prevalence of diabetes was higher in the TD group (31.15% vs. 24.51%, P = 0.032), whereas hypertension prevalence was comparable. Use of lipid-lowering medications was more frequent among men with TD (32.79% vs. 23.53%, P = 0.043).

**Table 1:** Demographic and clinical characteristics of men with and without TD.

Variable	TD (n=183)	Without TD (n=204)	P
Age (years) *	63.03 ± 4.80	61.69 ± 5.23	0.009 **^b^**
BMI (kg/m^2^)	28.50 ± 2.67	27.67 ± 2.77	0.003 **^b^**
Personal history			
Smoking, n (%)	54 (29.51)	57 (27.94)	0.734 **^c^**
Alcohol user, n (%)	65 (35.52)	69 (33.82)	0.726 **^c^**
Regular exercise, n (%)	65 (35.52)	84 (41.18)	0.253 **^c^**
Taking lipid-lowering drugs, n (%)	60 (32.79)	48 (23.53)	0.043 **^c^**
Hematologic parameters			
TG (mmol/L) *	2.01 (0.87)	1.72 (0.80)	<0.001 **^a^**
TC (mmol/L)	5.36 ± 0.61	4.88 ± 0.55	<0.001 **^b^**
HDL-C, (mmol/L)	0.94 ± 0.21	1.10 ± 0.27	0.011 **^b^**
LDL-C, (mmol/L)	3.06 ± 0.51	2.95 ± 0.53	0.036 **^b^**
RC, (mmol/L)	1.43 ± 0.74	0.83 ± 0.68	<0.001 **^b^**
SHGB, (nmol/l) *	34.60 (11.30)	47.25 (13.07)	<0.001 **^a^**
E2, (pg/ml)	34.50 ± 6.58	32.50 ± 6.98	0.183 **^b^**
FT, (ng/dL) *	3.22 (2.56)	6.54 (2.15)	<0.001 **^a^**
hs-CRP, (mg/L)	2.42 ± 1.50	1.72 ± 0.98	<0.001 **^b^**
IL-6, (µg/L)	2.83 ± 1.38	2.31 ± 1.09	0.001 **^b^**
Hypertension, n (%)	99 (54.10)	101 (49.51)	0.367 **^c^**
Diabetes, n (%)	63 (31.15)	50 (24.51)	0.032 **^c^**

Values are percentages or mean ± SD unless noted otherwise. *Values are presented as median with interquartile range. Regular exercise: Twice a week, at least 30 minutes each time.

TD, testosterone deficiency; BMI, Body Mass Index; TG, Triglyceride; TC, Total Cholesterol; HDL-C, high-density lipoprotein cholesterol; LDL-C, Low-density lipoprotein cholesterol; RC, Residual cholesterol; SHBG, Sex hormone-binding globulin; E2, estradiol; FT, free testosterone; hs-CRP, high-sensitivity C-reactive protein; IL-6, interleukin-6. ^a^ Mann-Whitney U test was used; ^b^ independent sample t tests; ^c^ chi-square test. Bold indicates P < 0.05.

Regarding lipid profiles, men with TD had higher TG [2.01 (0.87) vs. 1.72 (0.80) mmol/L], TC (5.36 ± 0.61 vs. 4.88 ± 0.55 mmol/L), LDL-C (3.06 ± 0.51 vs. 2.95 ± 0.53 mmol/L), and RC (1.43 ± 0.74 vs. 0.83 ± 0.68 mmol/L), together with lower HDL-C levels (0.94 ± 0.21 vs. 1.10 ± 0.27 mmol/L) (all P < 0.05). In addition, SHBG [34.60 (11.30) vs. 47.25 (13.07) nmol/L] and FT [3.22 (2.56) vs. 6.54 (2.15) ng/dL] were significantly lower in the TD group, whereas hs-CRP (2.42 ± 1.50 vs. 1.72 ± 0.98 mg/L) and IL-6 (2.83 ± 1.38 vs. 2.31 ± 1.09 µg/L) were higher; estradiol levels were similar between groups.

### Association between remnant cholesterol and testosterone deficiency

**Figure 2:**
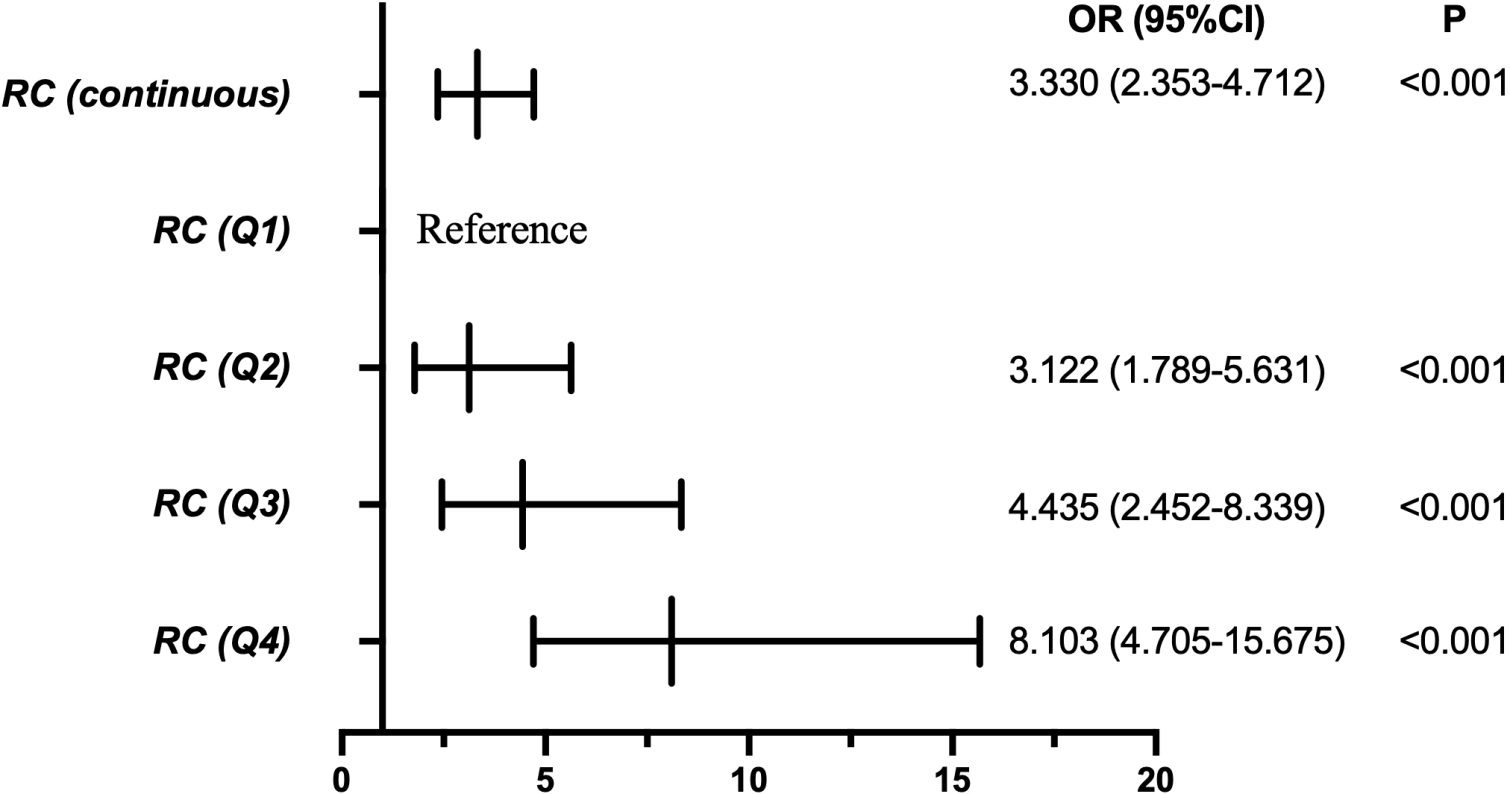
Multivariable logistic regression analysis showing the association between remnant cholesterol (RC) and testosterone deficiency (TD). RC was analyzed as both a continuous variable and quartiles (Q1–Q4), with the lowest quartile serving as the reference group.

As shown in **Table 2**; **Figure 2**, higher RC levels were independently associated with an increased risk of TD. In the fully adjusted model, RC analyzed as a continuous variable was significantly associated with TD (OR = 3.330, 95% CI: 2.353–4.712; P < 0.001). When RC was categorized into quartiles, a clear dose–response relationship was observed. Compared with participants in the lowest RC quartile (Q1), the odds of TD were significantly higher in Q2 (OR = 3.122, 95% CI: 1.789–5.631), Q3 (OR = 4.435, 95% CI: 2.452–8.339), and Q4 (OR = 8.103, 95% CI: 4.705–15.675) (all P < 0.001). These associations remained robust after adjustment for age, BMI, smoking status, alcohol use, regular exercise, lipid-lowering drug use, hypertension, diabetes, and TG levels.

**Table 2:** Multivariate logistic regression analysis for TD (TD vs. without TD).

Variable	Adjusted model		
	OR	95% CI	P
RC (continuous)	3.330	2.353-4.712	<0.001
RC (Quartiles)			
Q1 (<0.485)	Reference	Reference	Reference
Q2 (0.485-1.04)	3.122	1.789-5.631	<0.001
Q3 (1.04-1.595)	4.435	2.452-8.339	<0.001
Q4 (>1.595)	8.103	4.705-15.675	<0.001

Statistical Analysis: Adjusted model: adjusted for age, BMI, smoking status, alcohol user, regular exercise, taking lipid-lowering drugs, hypertension, diabetes, and TG.

TD, testosterone deficiency; BMI, Body Mass Index; TG, Triglyceride; RC, Residual cholesterol; Q, quartiles.

### Diagnostic performance of lipid parameters for identifying testosterone deficiency

**Figure 3:**
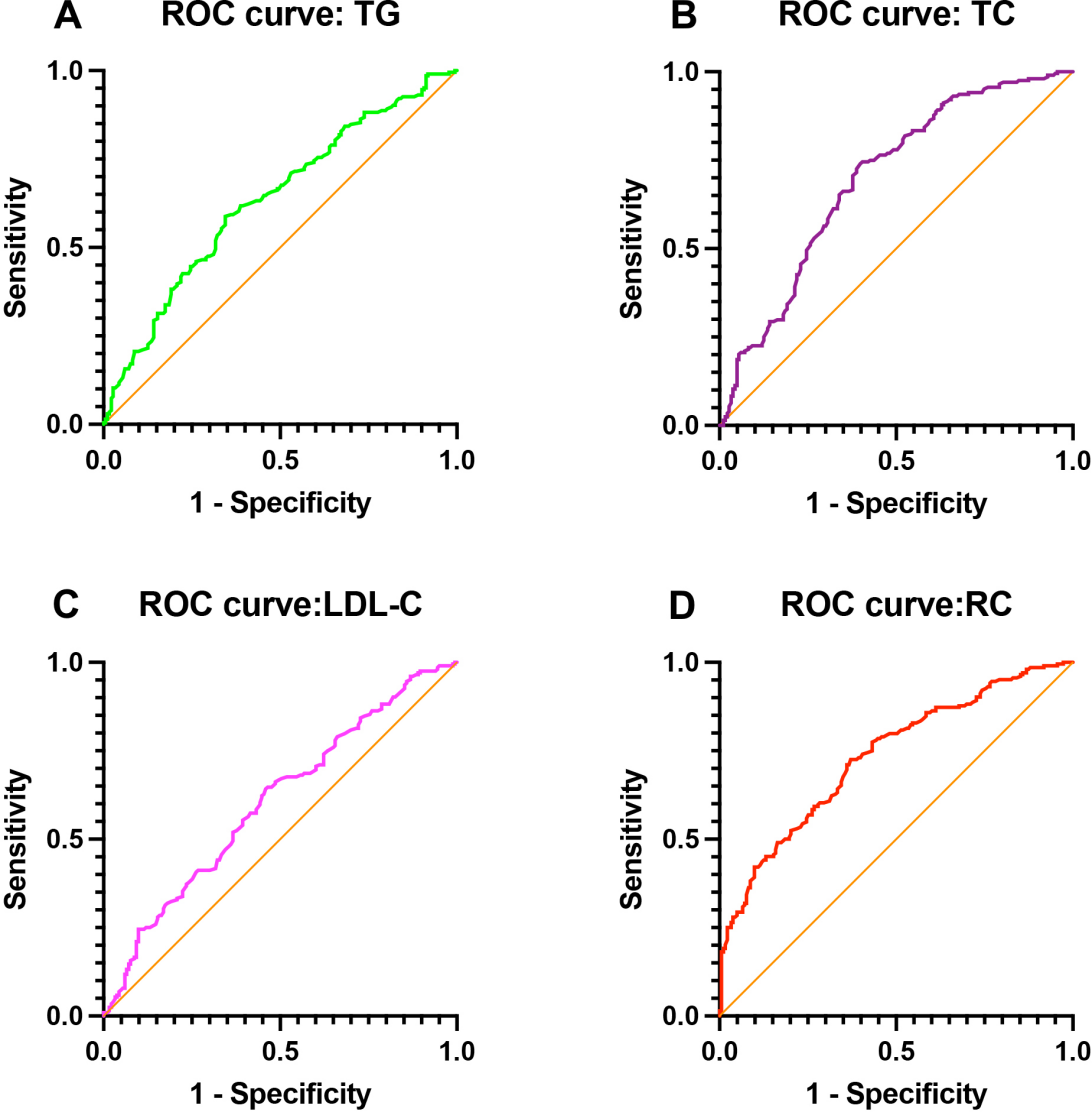
Receiver operating characteristic (ROC) curves evaluating the discriminative performance of : **(A)** triglycerides (TG), **(B)** total cholesterol (TC), **(C)** low-density lipoprotein cholesterol (LDL-C), and **(D)** remnant cholesterol (RC) for identifying testosterone deficiency.

The diagnostic performance of different lipid parameters for identifying TD was shown in **Table 3**; **Figure 3**. Among the evaluated lipid indicators, RC demonstrated the highest discriminative ability for TD, with an area under the curve (AUC) of 0.733 (95% CI: 0.684–0.782, P < 0.001). The optimal cut-off value for RC was 1.165 mmol/L, yielding a sensitivity of 62.8% and a specificity of 72.5%. In comparison, TC showed a moderate predictive performance with an AUC of 0.696 (95% CI: 0.643–0.749, P < 0.001), while TG and LDL-C exhibited lower discriminative ability, with AUC values of 0.634 (95% CI: 0.578–0.689) and 0.605 (95% CI: 0.549–0.661), respectively (all P < 0.001). Overall, RC outperformed traditional lipid parameters in identifying individuals with TD.

**Table 3:** Diagnostic performance of TG, TC, LDL-C, and RC for identifying TD.

Indicator	Optimal Cut-off (mmol/L)	Specificity	Sensitivity	AUC (95%CI)	P value
TG, mmol/L	1.805	0.588	0.656	0.634 (0.578-0.689)	<0.001
TC, mmol/L	5.175	0.740	0.601	0.696 (0.643-0.749)	<0.001
LDL-C, mmol/L	3.135	0.642	0.541	0.605 (0.549-0.661)	<0.001
RC, mmol/L	1.165	0.725	0.628	0.733 (0.684-0.782)	<0.001

TD, testosterone deficiency; TG, Triglyceride; TC, Total Cholesterol; LDL-C, Low-density lipoprotein cholesterol; RC, Residual cholesterol; AUC, Area Under the Curve; CI, Confidence Interval.

### Mediation analysis

**Figure 4:**
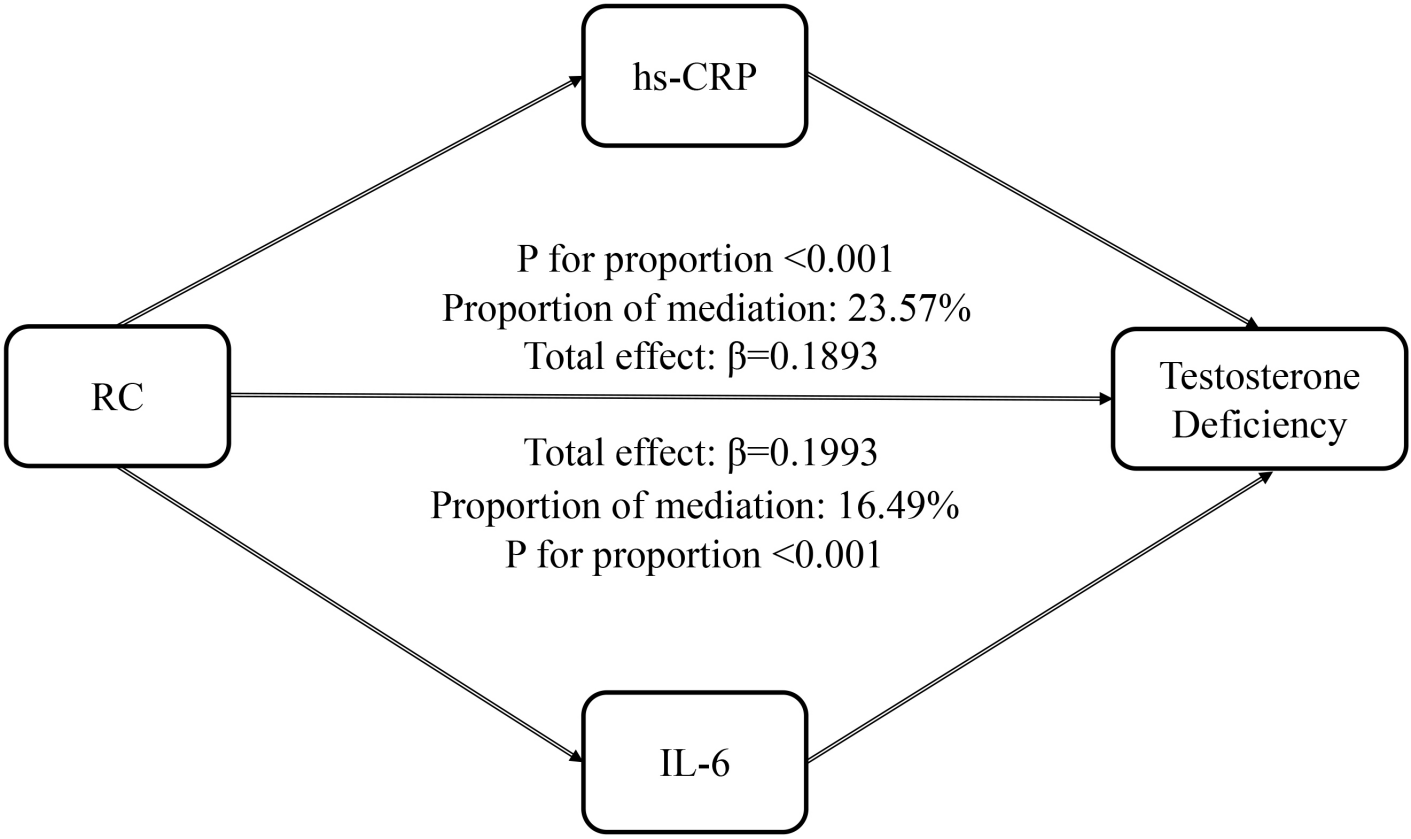
Mediation models illustrating the indirect effects of high-sensitivity C-reactive protein (hs-CRP) and interleukin-6 (IL-6) on the association between remnant cholesterol (RC) and testosterone deficiency.

The potential mediating roles of systemic inflammatory markers in the association between RC and TD were further examined using multiple mediation analysis (**Figure 4**). The total effect of RC on TD was statistically significant. When hs-CRP was included as a mediator, hs-CRP accounted for 23.57% of the total effect, with a significant indirect effect (P < 0.001). Similarly, IL-6 was identified as a significant mediator in the RC–TD association, explaining 16.49% of the total effect, with a significant indirect effect based on bootstrap analysis (P < 0.001). These findings indicate that systemic inflammation, as reflected by hs-CRP and IL-6 levels, partially mediates the association between elevated RC and TD.

## Discussion

In this multicenter cross-sectional study of middle-aged and older men, we observed a significant association between RC and TD. Higher RC levels were associated with increased odds of TD when analyzed as both a continuous variable and in quartiles, with a clear gradient across RC categories. These associations remained robust after adjustment for demographic factors, lifestyle variables, metabolic comorbidities, medication use, and TG levels, suggesting that RC may capture lipid-related information beyond traditional lipid parameters in relation to testosterone status. In addition, RC demonstrated a moderate discriminative ability for identifying TD and outperformed conventional lipid measures, including total cholesterol, triglycerides, and LDL-C. Furthermore, mediation analyses indicated that systemic inflammatory markers, specifically hs-CRP and IL-6, partially accounted for the association between RC and TD. Importantly, given the cross-sectional design of the present study, these findings should be interpreted as associative rather than causal, and the observed relationships do not establish temporal or mechanistic pathways.

Beyond TD, remnant cholesterol has been increasingly recognized as an important cardiometabolic risk factor. RC, carried in triglyceride-rich lipoproteins such as very-low-density lipoproteins, intermediate-density lipoproteins, and chylomicron remnants, plays a key role in the development of atherosclerotic cardiovascular disease (ASCVD) (25, 26). Large epidemiological and long-term cohort studies have demonstrated that elevated RC is associated with ASCVD risk independent of traditional lipid markers, including LDL-C and apolipoprotein B (27). Conversely, low testosterone levels have also been consistently linked to adverse cardiovascular outcomes, underscoring the close interplay between androgen status and cardiovascular health (5). Recent population-based studies using the NHANES, including our previous research, further reported that higher RC levels were associated with lower circulating testosterone concentrations and a higher prevalence of TD (18, 19). These findings provided initial epidemiological evidence supporting a link between RC and androgen status at the population level. The present study extends these observations by confirming a similar association in a real-world clinical setting, where TD was defined according to guideline-based criteria incorporating both biochemical measurements and clinical symptoms. Taken together, the consistent associations observed across NHANES-based analyses and our multicenter clinical cohort suggest that RC and TD may coexist within a broader cardiometabolic context. Although causality cannot be inferred from cross-sectional data, these findings highlight the potential relevance of considering lipid abnormalities characterized by elevated RC when evaluating men with TD, and vice versa, and support the need for longitudinal studies to clarify temporal relationships and underlying mechanisms.

Several mechanisms may underlie the observed association between RC and TD, with metabolic dysfunction and chronic low-grade inflammation likely playing important roles. Low testosterone levels have been recognized as a component of metabolic syndrome (28) and are associated with visceral fat accumulation, insulin resistance, and increased metabolic risk (29–31). Adipose tissue may further aggravate androgen deficiency by sequestering circulating testosterone, promoting its conversion to estradiol, and suppressing hypothalamic signals that drive testosterone production (8). Consistent with this, previous studies have shown that elevated RC levels are associated with adverse visceral fat accumulation and insulin resistance, and that body mass index and insulin resistance may partially mediate the relationship between RC and low testosterone (32). From another perspective, elevated RC may contribute to a proinflammatory milieu that adversely affects testosterone synthesis. Excess remnant lipoproteins can promote chronic low-grade inflammation through lipid metabolism–related pathways, endothelial penetration, and macrophage activation (33, 34). Proinflammatory cytokines, such as IL-6 and tumor necrosis factor-α, have been shown to impair hypothalamic–pituitary–gonadal axis function and directly inhibit Leydig cell steroidogenesis (35, 36). Cholesterol overload may also induce cellular stress and oxidative damage, further suppressing testosterone production (37). Taken together, these observations suggest that the association between RC and TD may reflect the interplay of lipid abnormalities, adiposity, insulin resistance, and inflammation; however, given the cross-sectional design, these mechanisms remain speculative and require confirmation in longitudinal and experimental studies.

Beyond its role as a lipid fraction, RC may reflect a broader lipo-inflammatory phenotype, characterized by the interaction between lipid accumulation and immune activation. Remnant lipoproteins are enriched in cholesterol and bioactive lipid components, including oxidized lipids, which can promote endothelial dysfunction and stimulate inflammatory signaling pathways (38). Emerging evidence suggests that lipid burden and immune activation are closely interconnected in metabolic disorders. For example, studies in familial hypercholesterolemia have demonstrated that markers of innate immune activation, such as the neutrophil-to-lymphocyte ratio (NLR), are associated with elevated LDL-C levels and subclinical atherosclerosis (39), highlighting the presence of an intrinsic lipo-inflammatory state even in the absence of overt CVD. Similarly, in high-risk dyslipidemia populations, elevated lipoprotein(a) (Lp(a)) levels have been associated with persistent inflammatory and atherothrombotic activity, even in the presence of high-intensity statin therapy (40). In this context, elevated RC may not only reflect dyslipidemia but also indicate a systemic inflammatory environment capable of influencing various metabolic processes, including Leydig cell steroidogenesis. Thus, RC in men with TD may represent not only a marker of lipid dysregulation but also an indicator of ongoing immune-metabolic activation, which may contribute to endocrine dysfunction and cardiovascular risk.

From a clinical perspective, the observed association between remnant cholesterol and testosterone deficiency may have implications for risk assessment in middle-aged and older men. RC is a routinely available lipid parameter that can be readily derived from standard lipid profiles, and its elevation may help identify individuals who also present with a higher likelihood of testosterone deficiency in clinical settings. Conversely, men evaluated for testosterone deficiency often exhibit concurrent metabolic abnormalities, and consideration of RC may provide additional information beyond traditional lipid markers when assessing their cardiometabolic profile. Importantly, these findings do not imply that RC should be used as a diagnostic or therapeutic target for testosterone deficiency. Rather, they highlight the potential value of integrated metabolic and hormonal evaluation in men presenting with symptoms of androgen deficiency. Future longitudinal and interventional studies are needed to determine whether combined assessment of lipid abnormalities characterized by elevated RC and androgen status can improve risk stratification and guide personalized management strategies.

Several limitations of the present study should be acknowledged. First, the cross-sectional design precludes inference of temporal or causal relationships between RC and TD. Therefore, it remains unclear whether elevated RC contributes to the development of TD or whether hormonal alterations influence lipid metabolism. In addition, although mediation analyses suggested that systemic inflammatory markers may partially explain the observed association, mediation models based on cross-sectional data cannot establish causal mediation and should be interpreted cautiously. Second, despite adjustment for multiple demographics, lifestyle, and metabolic variables, residual confounding cannot be completely excluded. Potential factors such as dietary habits, physical activity intensity, socioeconomic status, psychological stress, and sleep quality were not comprehensively assessed and may influence both lipid metabolism and testosterone levels. Third, lipid profiles, inflammatory markers, and hormone levels were measured at a single time point. Given the known intra-individual variability in these biomarkers, single measurements may introduce misclassification and may not fully capture long-term metabolic or hormonal status. Fourth, remnant cholesterol was calculated using the widely accepted formula (RC = TC − HDL-C − LDL-C) rather than directly measured remnant lipoprotein concentrations. Although calculated RC has been extensively used in epidemiological studies and clinical research, direct measurement of remnant lipoproteins may provide greater analytical precision. Fifth, the study population consisted of Chinese men aged 50–75 years recruited from urology and andrology outpatient clinics, which may limit the generalizability of the findings to younger individuals, other ethnic groups, or community-based populations. Finally, although RC demonstrated a moderate discriminative ability for identifying testosterone deficiency, its predictive performance alone is insufficient for diagnostic purposes. Instead, RC may serve as a complementary metabolic indicator within a broader clinical assessment.

## Conclusion

In this multicenter cross-sectional study, higher RC levels were associated with an increased prevalence of TD among middle-aged and older men, and RC showed greater discriminative ability than traditional lipid parameters. These findings suggest that RC may serve as a useful marker in the metabolic assessment of men with TD, while further longitudinal studies are warranted to clarify temporal relationships and underlying mechanisms.

## Data Availability

The original contributions presented in the study are included in the article/supplementary material. Further inquiries can be directed to the corresponding authors.
